# Carving of Block-Teeth

**Published:** 1895-05

**Authors:** F. A. Coney

**Affiliations:** Doylestown, Pa.


### CARVING OF BLOCK-TEETH.[1]

BY F. A. CONEY, D.D.S., DOYLESTOWN, PA.

Hand-carved blocks have the merit of being the most natural-looking and at the same time, when well mounted, the strongest artificial teeth made. When the best results are desired, and neither time nor cost are objects, carved blocks are the best recourse of the dentist.

It is here that the carver has an opportunity to carry out his conception of a very difficult and peculiar case. The teeth can be placed in any position or inclined at any angle deemed desirable. The natural expression of a carved block is seen in the bolder curves and contours made by the skilful hand, which it is impossible to produce from brass moulds.

The following is a description, in detail, of the carving process in general use from about the year 1850 to date:

The first step is to get a perfect impression, and a bite for an upper or lower, whichever space the piece is intended for. The simplest and shortest method of getting a bite for small partial cases is the following:

Take a roll of beeswax—or modelling composition, which I prefer—from one to two inches long, according to the number of missing teeth, and about three-fourths of an inch thick. Soften in hot water, bend into a semicircle, and press it against the teeth on each side of the space or spaces requiring substitutes. Then direct the patient to bite through the wax until the cutting-edge and cusps touch and occlude naturally. After pressing the wax or modelling composition against the labial or buccal surfaces of the teeth, carefully remove it from the mouth, and harden it in cold water.

Now, then, take water and plaster and mix it quite thin. First fill the imprints of the teeth in the wax or modelling composition, and work the plaster into said imprints. As the plaster stiffens build it up and extend it back over the palatine surface, and about one-half or two inches beyond said impression. That will give the upper part of the model. Trim it to the desired shape to handle conveniently. Now sandarach or shellac the plaster exposed to view, then oil the model and bite with sweet oil. The next step is

[1] Read before the Odontological Society of Pennsylvania, December 8, 1894.

to run the lower part of the model with plaster. When plaster is set, put said model in hot water to soften the wax or modelling composition.

When soft take it apart, and you have the model of the mouth to carve your block by.

The next step is to enlarge the cast. If it is to be a block of two teeth, cut from the approximate surface of each tooth of both sides of space to be filled about one-half of a sixteenth of an inch. This will give the shrinkage for said block.

For a block of four teeth you proceed in the same manner as for a block of two teeth, with the exception that you take from the approximate surfaces one-sixteenth of an inch from each tooth, thus allowing a greater shrinkage for a block of four teeth than for a block of two teeth.

For a block of six teeth, you proceed in the same manner as you did for the block of two or four teeth, allowing, of course, for a greater shrinkage, according to the case you have in hand.

Now prepare in a small tin cup spermaceti, melting over a spirit lamp until it becomes liquid. Add enough Chinese or red vermilion to color it to a cherry red. Then take the liquid spermaceti and paint with a camel's-hair brush your impression model, the space between the canines. If it is to be a block of four, also paint the canines. For a block of six do the same as for a block of four, in the same manner for all models which are used for carving of teeth.

Apply with a camel's-hair brush sweet oil over the antagonizing model or bite, so the bite will separate from the body without drawing it.

I use Lukens's body, prepared by S. S. White. It is the strongest body known, and fuses at the highest heat. This body I mix in a porcelain bowl or wedge-wood mortar by adding water to it to make it the consistency of putty. It is now ready to be packed or worked into the model. When the model is full of body, dry out the body on a muslin cloth so as to absorb the moisture which arises on the surface; it also makes the body more solid and firm. It is then ready to begin carving.

The carving instruments are known as a string-bow, carving-knives of different shapes, one bone spatula, pin tweezers, and camel's-hair pencils.

Before commencing this process the size and shape of the teeth must be decided upon, the perfect form of the ideal teeth must be in the mind's eye. The width of the teeth is then marked off on the block, beginning at the central or median line, the desired width and height. With the straight carving-knife, and by cutting an inverted V-shape between the teeth, the necks are carved in a semicircular groove, not deep; have them to incline towards the centre equally on both sides.

The model is now reversed, and the points of the gum between the teeth are carved down, and then the body is added to each tooth to bring it up to the desired shape and size, and to look as natural as possible. The gum is then carved the desired thickness, and also to give shape to the block. The palatine surface of each tooth must be carved so as to antagonize with the opposing teeth. A recess must be made in the block for the pins. The pins are then inserted, and body worked around them.

Now comes the difficult part. To take the block off the model, heat the block over the flame of an alcohol-lamp (a large one preferred) until the spermaceti is all melted from underneath the block. The greater part of the spermaceti will be absorbed in the body. It will then be loose on the model. Now drop the block on a piece of cotton, and be very careful not to let it have too great a fall. While the block is cooling, which takes about five minutes, take a slide and put a sufficient amount of kaolin, say about one inch in height and enough in width to lay the block upon. Pick up the block with the thumb and forefinger and place it with the palatine surface, or pin side, on the kaolin. Now make about half a dozen cone-shaped pieces with the body, about one inch high. These are to be used as trial pieces.

The block is now ready for biscuiting. Biscuiting is the hardening of the block in a red-hot muffle. The furnace used for biscuiting and baking is an ordinary two-muffled furnace, purchased at any of the dental depots. First put wood in the furnace, then put the slide containing the block in the back part of the lower muffle, leaving the door open for the escape of the smoke which arises from the grease in the block, then start the wood to burning and put on about two or three bucketfuls of coal. Now, when looking at the block in the muffle, the teeth or block will be found to have turned black, caused by the grease in the block being burnt out.

As the fire increases, the muffle becomes red on the inside; the teeth will then resume their former shade; at this stage close the muffle-door, and wait until the muffle comes to a bright-red heat; then take out a trial piece and with a penknife cut to see if it is as hard as pipe-clay. If so, it is time to remove the slide containing the block from the muffle; check the fire by removing the stoppers on the sides and top of the furnace. When the block is cool remove it from the slide, brush off the kaolin, and transfer the block to the model. It is now ready for enamelling. Enamels are technically called neck-, point-, stain-, and gum-enamels.

The enamels are then applied to the block with camel's-hair pencils by holding the block-teeth upward. Enamels should be mixed with clean water in a small glass or porcelain cup, making a cream-like solution. The neck-enamel is applied first at the neck of the tooth, extending half-way up the teeth towards the cutting-edge or point. The neck-enamels vary in color from bright yellow to dark brown. Now apply the point-enamel on the cutting-edge of the tooth, and bring the point-enamel down over the neck-enamel, so as to overlap or coalesce. The point-enamels vary from white to different tints of bluish gray and yellow. The gum-enamel is applied to the points between the teeth with the carving-knife; the rest of the gum-enamel is put on the block with a camel's-hair pencil, care being taken to place the gum-enamel close to the necks of the teeth, but not to overlap the neck-enamel. It is not best to make the gum-enamel very smooth; it fuses at a little lower heat than the point- and neck-enamels, and is apt to become glassy.

The festoons around the necks of the teeth should be ridged, so as to make them more natural-looking and give them a certain prominence and individuality of their own; so the teeth will look as if they had grown out of the gum naturally. Many blocks are ruined by not having the gum-enamel applied properly.

After the enamelling is all done, prepare a slide the same as was done for biscuiting, with this exception,—instead of laying the block pin-side down on the kaolin, place the block perpendicular, the cutting-edge of the teeth upward; enamel your trial pieces at the points with gum-enamel, and place them on the slide in front of the block. Now all is ready for the final baking.

Place in your furnace the side stoppers, add coal to the fire, and fill the furnace with fresh coal; then put in the upper stopper, close all openings around stoppers with fire-clay mixed with water. When the lower muffle is at a white heat the slide is then grasped with a pair of long tongs, the ends of which are flattened for this purpose. The slide is lifted and held before the muffle for about two or three minutes, or until the block is thoroughly dry, then put the slide into the fore part of the lower muffle and gradually move it towards the back part of the muffle. When you reach the baking part, which can be distinguished by the intense glow or white heat, close the muffle-door, and give the block about ten minutes' time; then take out a trial piece with the tongs, and examine to see if the trial piece is fused or glazed enough; if not, bake about three minutes longer, take out another trial piece, and if it is fused and glazed enough, the block is done.

Now take the stopper out of the upper muffle; draw the slide from the lower muffle and pass it quickly into the back part of the upper muffle; close up the muffle with the stopper and close all openings with fire-clay; draw the fire and let block remain in the annealing or cooling muffle until cold. When cold the block is ready for grinding, which all blocks require more or less. Take a new impression and make a cast or model to grind the block to fit. To facilitate the grinding, the prominent parts of the cast are coated with a mixture of red vermilion and sweet oil which will spot the under side of the block and show the exact place to be ground off to make the block fit solidly on cast. After this is done it is ready to be mounted upon any base the operator may wish.

